# EZH2抑制剂与吉非替尼联合应用在EGFR-TKIs耐药肺癌细胞中的作用研究

**DOI:** 10.3779/j.issn.1009-3419.2019.05.01

**Published:** 2019-05-20

**Authors:** 颢 宫, 茵 袁, 永文 李, 洪兵 张, 颖 李, 伟婷 李, 攀 王, 睿峰 施, 超 刘, 力元 崔, 红雨 刘, 军 陈

**Affiliations:** 1 300052 天津，天津医科大学总医院肺部肿瘤外科 Department of Lung Cancer Surgery, Tianjin 300052, China; 2 天津市肺癌研究所，天津市肺癌转移与肿瘤微环境重点实验室 Tianjin Key Laboratory of Lung Cancer Metastasis and Tumor Microenvironment, Tianjin Lung Cancer Institute, Tianjin Medical University General Hospital, Tianjin 300052, China

**Keywords:** 肺肿瘤, EZH2, EZH2抑制剂, 吉非替尼, 获得性耐药, Lung neoplasms, EZH2, EZH2 inhibitor, Geftinib, Acquired resistance

## Abstract

**背景与目的:**

肺癌是危害身体健康的常见恶性肿瘤之一。随着表观遗传学的发展，研究者发现Zeste基因增强子同源物基因2（enhancer of Zeste homolog 2, EZH2）在肺癌中高表达且其表达量与预后息息相关，与此同时EZH2抑制剂也被发现能够提升肿瘤细胞对多种抗肿瘤药物的敏感性。本研究的目的在于探讨EZH2抑制剂与吉非替尼（gefitinib）联合应用，对耐吉非替尼细胞的增殖、凋亡及迁移的影响。

**方法:**

以PC9吉非替尼敏感细胞和PC9/AB2吉非替尼耐药细胞为研究对象，应用CCK-8及EdU实验检测联合用药对于细胞活性及增殖的影响；通过划痕及Transwell小室检测联合用药对于细胞迁移能力的影响；并应用流式细胞学检测联合用药对于细胞凋亡的影响；并通过Western blot观察联合用药对EZH2及表皮生长因子（epidermal growth factor receptor, EGFR）信号通路相关蛋白的作用。

**结果:**

在吉非替尼耐药细胞PC9/AB2细胞中，联合EZH2抑制剂GSK343与吉非替尼能够显著抑制耐药细胞的活性，降低细胞的迁移能力并诱导耐药细胞的凋亡。同时，联合用药也能够显著抑制EZH2表达及EGFR蛋白的磷酸化。

**结论:**

EZH2抑制剂GSK343与吉非替尼联合应用能够克服PC9/AB2对吉非替尼的耐药作用，此研究也提示协同治疗在肺癌EGFR-TKIs耐药逆转中具有一定的作用。

## Abstract (en)

**Background and objective:**

Lung cancer is one of the common malignant tumors that impair human health. With the development of epigenetics, the researchers found that enhancer of Zeste homolog 2 (EZH2) is highly expressed in lung cancer tissue and its expression is closely related to the prognosis. EZH2 inhibitor can also enhance the sensitivity of tumor cells to a variety of anti-tumor drugs. The purpose of this study is to investigate the effect of combination of EZH2 inhibitor and gefitinib on the proliferation, apoptosis and migration of Gefitinib-resistant lung cancer cells.

**Methods:**

PC9 and PC9/AB2 cells were used for this study. CCK-8 and EdU experiment were used to detect combined treatment on cell viability and proliferation activity; Wound healing assay and Transwell chamber experiment were used to determine the effects of combination therapy on cell migration ability; Flow cytometry was used to detect the effect of combination therapy on EZH2 and apoptosis; Western blot was used to observe the effect of combination therapy on epidermal growth factor receptor (EGFR) signaling pathway-related proteins expression.

**Results:**

In gefitinib-resistant cell line PC9/AB2, gefitinib combined with EZH2 inhibitor GSK343 can significantly inhibit cell viability, reduce cell migration and increase cell apoptosis. At the same time, combination therapy can significantly inhibit the expression of EZH2 and phosphorylation EGFR proteins.

**Conclusion:**

The combination of EZH2 inhibitor GSK343 and gefitinib sensitize PC9/AB2 cell to gefitinib response. This study also suggests that synergistic therapy plays a role in the reversal of EGFR-tyrosine kinase inhibitor (EGFR-TKIs) resistance in lung cancer.

肺癌是我国最常见的恶性肿瘤之一，具有较高的发病率及病死率，并且发病率逐年增高，居我国一些大中城市各种恶性肿瘤的首位。其中非小细胞肺癌（non-small cell lung cancer, NSCLC）占所有肺癌的80%^[1]^。早期肺癌治疗主要以手术为主，但由于早期症状不明显等原因，30%-40%的患者在确诊时已经错过手术的最佳时期，且肺癌术后也有较高的复发率与转移率^[2]^。对于已经错过手术指征的患者临床上大多采用以放化疗为主的治疗方案，然而现放化疗的已进入瓶颈期，无法得到进一步的治疗突破，且部分肿瘤的放化疗效果并不好。

靶向治疗是肺部肿瘤治疗的一个新的突破口。其中，针对表皮生长因子受体酪氨酸激酶抑制剂（epidermal growth factor receptor tyrosine kinase inhibitor, EGFR-TKIs）的分子靶向药物的研究和开发是目前肺癌靶向治疗最主要的热点。最新的美国国立综合癌症网络（National Comprehensive Cancer Network, NCCN）指南中也明确指出：对具有*EGFR*敏感突变的肺癌患者，应用EGFR-TKIs靶向药物，纳入了一线治疗方案中^[3]^。EGFR-TKIs类药物的具有疗效好、副反应小、服药方便等特点，在临床上广受患者的欢迎。自IPASS研究以来，多项Ⅲ期临床试验证实第一代TKI治疗*EGFR*突变晚期NSCLC患者疗效的确切性。第一代TKI类药物（吉非替尼、厄洛替尼）作为一线治疗*EGFR*突变阳性的NSCLC的疗效明显优于含铂双药化疗^[4]^。CONVINCE Ⅲ期临床实验结果表明：在*EGFR*突变的晚期肺腺癌患者中，应用EGFR-TKIs类药物埃克替尼比培美曲赛联合铂类一线治疗后培美曲塞维持的疗效更为明显^[5]^。对于晚期*EGFR*突变的NSCLC患者一线采用一代EGFR-TKI类药物治疗，不管是有效率还是无症状生存期（progression-free survival, PFS）均明显优于以铂类为基础的化疗，但应用第一代EGFR-TKIs治疗会面临一个严峻的问题：绝大多数肺癌患者会在治疗后1年左右出现不同程度的耐药，导致疾病的复发和治疗的失败。因此，针对EGFR-TKIs耐药及逆转耐药研究、联合应用其他类药物延缓耐药周期成为目前研究的热点。

随着分子生物学的发展，研究人员发现肿瘤组织中除会出现特定基因位点改变及异常信号通路激活外，还会出现一些特殊的表观遗传性状的改变，如DNA的甲基化、磷酸化或乙酰化^[6-9]^。EZH2是一个重要的表观遗传调控基因，具有组蛋白甲基转移酶（histone methyltransferase, HMT）活性，能够对组蛋白H3第27位赖氨酸三甲基（H3K27me3）催化从而抑制靶基因的表达^[7]^。*EZH2*基因在NSCLC中高表达，且表达水平与患者的预后密切相关。同时研究也证实应用EZH2抑制剂能有效地降低NSCLC的侵袭和迁移的能力，并能诱导肿瘤细胞凋亡^[10, 11]^。可见EZH2抑制剂在肺癌治疗领域具有良好的应用前景和临床价值。同时EZH2也有望成为治疗NSCLC治疗的新的靶点，本研究主要探讨EZH2抑制剂与吉非替尼联合，是否能增加肺癌细胞对吉非替尼敏感性，同时联合应用GSK343+吉非替尼对肺癌细胞的生物学行为的影响。

## 1 材料与方法

### 1.1 细胞株及主要仪器

PC9及其耐药亚克隆细胞系PC9/AB2由本研究所保存。PC9具有EGFR外显子19（E746-A750del）的缺失突变，为吉非替尼敏感细胞系。PC9/AB2细胞是通过PC9细胞长期暴露于吉非替尼建立出的吉非替尼耐药细胞系，在培养过程中在培养基中持续加入10 μmol/L的吉非替尼以维持其耐药性。RPMI-1640培养基、胎牛血清、0.25%胰蛋白酶-EDTA购自GIBCO公司（GIBCO-BRL, Grand Island, NY）EZH2抑制剂GSK343、吉非替尼（gefitinib）购自Selleck，CCK-8细胞增殖试剂盒购自碧云天生物技术公司，EdU试剂盒购自锐博生物公司，细胞凋亡试剂盒购自BD公司，P-EGFR抗体购自Abcam公司，EGFR抗体、EZH2抗体、Histone H3、H3K27me3抗体来自Cell Signaling Technology公司（Danvers, MA, USA），β-actin抗体购自Sigma公司（Kansas, Missouri, USA），流式细胞仪购自Beckman coulter公司（CA, USA）。

### 1.2 细胞培养及药物处理

PC9及PC9/AB2细胞用含10%胎牛血清（fetal bovine serum, FBS）的RPMI-1640培养，将细胞置于10 cm细胞培养皿，放入37 ℃、5%CO_2_饱和湿度的培养箱中，直至细胞处于对数生长期，用0.25%胰蛋白酶-EDTA消化液进行细胞传代，GSK343、gefitinib溶于于二甲基亚砜（dimethyl sulphoxide, DMSO）溶液中。根据实验需求处理细胞。

### 1.3 CCK-8分析联合用药对耐药细胞活性的影响

采用CCK-8试剂盒分析EZH2抑制剂与吉非替尼对PC9/AB2细胞活性的影响：取对数期生长期细胞，按每孔5×10^3^细胞铺于96孔板上，置于细胞孵箱中过夜培养，待细胞贴壁后，进行加药处理，处理时间为48 h。48 h后，取出96孔板，每孔加入20 μL的CCK-8溶液，继续37 ℃避光培养1 h，用酶标仪测各孔的吸光值（450 nm）。取各个复孔的平均值，药物抑制率计算公式：（未加药组的吸光值-实验组吸光值）/未加药组吸光值×100%，最终得出GSK343及gefitinib的IC_50_浓度及抑制率曲线，每组实验重复3次。

### 1.4 EdU检测DNA合成

EdU（5-ethynyl-2-deoxyuridine）是一种胸腺嘧啶核苷类似物，能够在细胞增殖时期代替胸腺嘧啶（T）渗入正在复制的DNA分子中，并能够与Apollo^®^荧光染料发生特异性反应，快速检测细胞DNA合成。我们将处于对数期的细胞用胰酶消化转移至96孔板，每孔细胞数为5×10^3^个。按实验要求向96孔板的细胞进行加药处理，加药处理后按照EdU试剂盒说明书对细胞进行不同处理。

### 1.5 细胞划痕实验

取对数生长期的PC9/AB2细胞铺在6孔板上，放入37 ℃，5% CO_2_饱和度的培养箱中，培养直至细胞长至90%密度，用无菌的免酶枪头在六孔板中央划痕，用PBS清洗去除悬浮细胞，随后按照实验要求加入不同处理组及阴性对照组，放入细胞孵箱培养，于0 h、16 h、32 h置于显微镜下观察拍照。上述实验独立重复3次。

### 1.6 Transwell小室实验

迁移实验：应用不同药物处理对数期的PC9/AB2细胞，处理时间为48 h。将提前处理好的4组细胞按照细胞1×10^4^个铺于上室并加入无血清的细胞培养基，混合液体总量为200 μL，于下室加入600 μL完全培养基，置于37 ℃、5%CO_2_恒温培养箱，培养12 h。取出小室用棉签小心拭去上室细胞，4%多聚甲醛固定10 min，用结晶紫室温染色25 min，并用PBS将多余染色液洗干净，显微镜下观察下室穿过的细胞，选取5个随机视野进行拍照计数。计算平均每个视野的细胞数，通过计算穿过的细胞数来评价细胞的迁移能力。每组实验独立重复3次。

### 1.7 流式细胞仪检测联合用药对PC9/AB2凋亡的作用

取对数生长期的细胞，按细胞浓度5×10^6^/孔，铺于6孔板上，按照实验要求对细胞进行分组处理。待处理后收集细胞，按照凋亡试剂盒的具体说明书对细胞进行PI染色，随后应用流式细胞仪上机检测。

### 1.8 蛋白质免疫印迹实验（Western blot, WB）

提取不同加药处理的细胞蛋白，应用PMSF（phenylmethanesulfonyl fluoride）+RIPA（radio immunoprecipitation assay）于冰上裂解30 min，BCA法测量蛋白浓度，加入SDS-PAGE蛋白缓冲液混匀置于100 ℃金属浴加热变性。取30 μg变性后的蛋白上样，80 V电泳2 h，120 V转膜80 min，应用5%脱脂牛奶/BSA封闭1 h。加入EZH2、EGFR、P-EGFR、Histone H3、H3K27me3、β-actin蛋白一抗，4 ℃过夜孵育，次日用TBST清洗后，加入对应二抗，显色曝光。

### 1.9 统计学方法

应用graphpad prism 6.0软件统计分析作图，实验中组间比较采用独立样本*t*检验，*P*值取双侧检验，*P* < 0.05为统计学有差异。划痕结果采用Image J 1.8.0版软件进行分析处理。

## 2 结果

### 2.1 应用EZH2抑制剂GSK343能够显著增强PC9/AB2细胞对吉非替尼的敏感性

对于单药作用，我们应用GSK343及吉非替尼处理细胞，各组药物分别按浓度梯度0 μmol/L、2 μmol/L、4 μmol/L、8 μmol/L、16 μmol/L、32 μmol/L、64 μmol/L、128 μmol/L依次递增，每组设4个复孔，每孔培养基液体为200 μL。应用CCK-8分别检测PC9及PC9/AB2对吉非替尼的敏感性。如图 1A所示，PC9的吉非替尼的IC_50_为2.73 μmol/L，而PC9/AB2 IC_50_=30.27 μmol/L，表明PC9/AB2对于吉非替尼耐药性远超敏感型PC9细胞（*P* < 0.01）。PC9和PC9/AB2的GSK343的IC_50_分别为16.45 μmol/L和18.14 μmol/L，二者统计学没有显著差异（*P*=0.519）（图 1B）。

**1:**
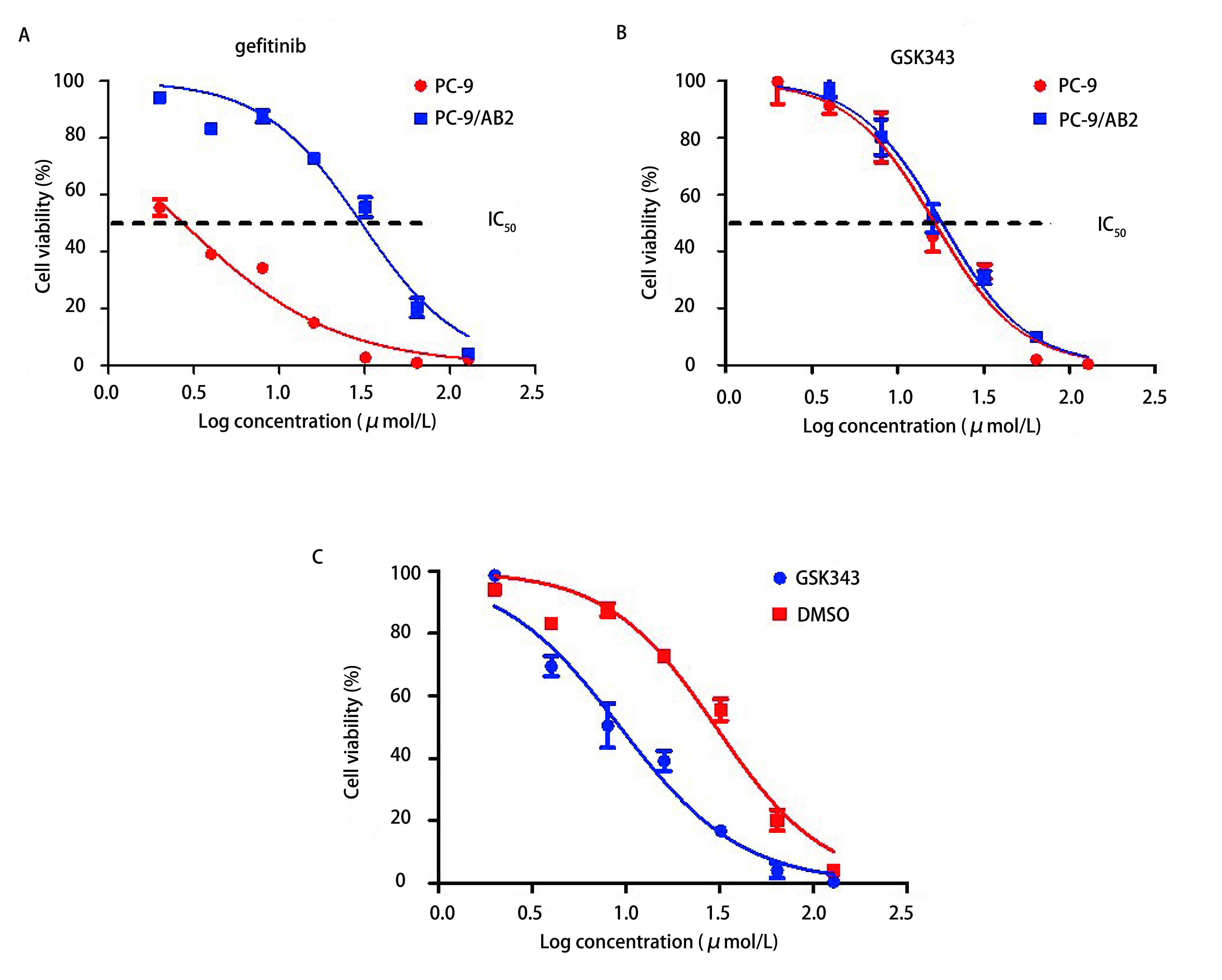
应用EZH2抑制剂GSK343能够显著增强PC9/AB2细胞对吉非替尼的敏感性。A：PC9及PC9/AB2对不同浓度吉非替尼的细胞活性曲线；B：PC9及PC9/AB2对不同浓度GSK343的细胞活性曲线；C：联合使用GSK343+吉非替尼能够增强耐药细胞对吉非替尼的敏感性。 EZH2 inhibitor GSK343 can significantly enhance the sensitivity to gefitinib of PC9/AB2 cells. A: Cell viability curves for PC9 and PC9/AB2 treated with different concentrations of gefitinib; B: Cell viability curves for PC9 and PC9/AB2 treated with different concentrations of GSK343; C: Combined use of GSK343+gefitinib can enhance the sensitivity to gefitinib of lung cancer cells.

为了进一步观察加入EZH2抑制剂GSK343后PC9/AB2细胞对于吉非替尼的敏感性，我们将细胞分为两组即：DMSO组（DMSO加药量等于溶解GSK343药物所需的DMSO量）与GSK343组（GSK343加药量我们选取GSK343 1/2 IC_50_即9 μmol/L），分别采用不同浓度梯度吉非替尼：0 μmol/L、2 μmol/L、4 μmol/L、8 μmol/L、16 μmol/L、32 μmol/L、64 μmol/L、128 μmol/L处理PC9/AB2细胞。如图 1C所示，加入GSK343后吉非替尼的IC_50_为9.327 μmol/L，较DMSO组30.27 μmol/L明显下降（*P* < 0.01），结果提示加入GSK343后PC9/AB2细胞对于吉非替尼的敏感性增加。

### 2.2 联合应用GSK343+吉非替尼能显著抑制PC9/AB2细胞的活性和增殖

为了进一步检验联合用药对PC9/AB2耐药细胞的作用效果，我们将实验处理组设定为4组即：NC组、GSK343组（9 μmol/L）、gefitinib组（15 μmol/L）、G+g组（9 μmol/L GSK343+15 μmol/L gefitinib），处理时间为48 h。如图 2A所示，CCK-8细胞活性实验结果显示：与NC对照组比较，GSK343、gefitinib、G+g的细胞活性分别为（85.31±2.47）%、（69.03±5.93）%、（31.29±1.23）%，提示单用GSK343及gefitinib能够抑制细胞的活性(*P*均 < 0.01)，而联合用药较单独用药对耐药细胞的抑制效果更明显（*P*均 < 0.01）。EdU结果显示: GSK343组、gefitinib组及G+g组阳性细胞数分别为（45.02±2.47）%、（41.62±3.98）%、（1.75±2.06）%，明显低于阴性对照组（NC）（52.03±3.87）%，*P*值分别为0.02、0.01、 < 0.01，提示GSK343、gefitinib对于细胞的增殖均有一定抑制作用，但两药合用对于细胞增殖抑制效果更为明显（图 2B）。

**2:**
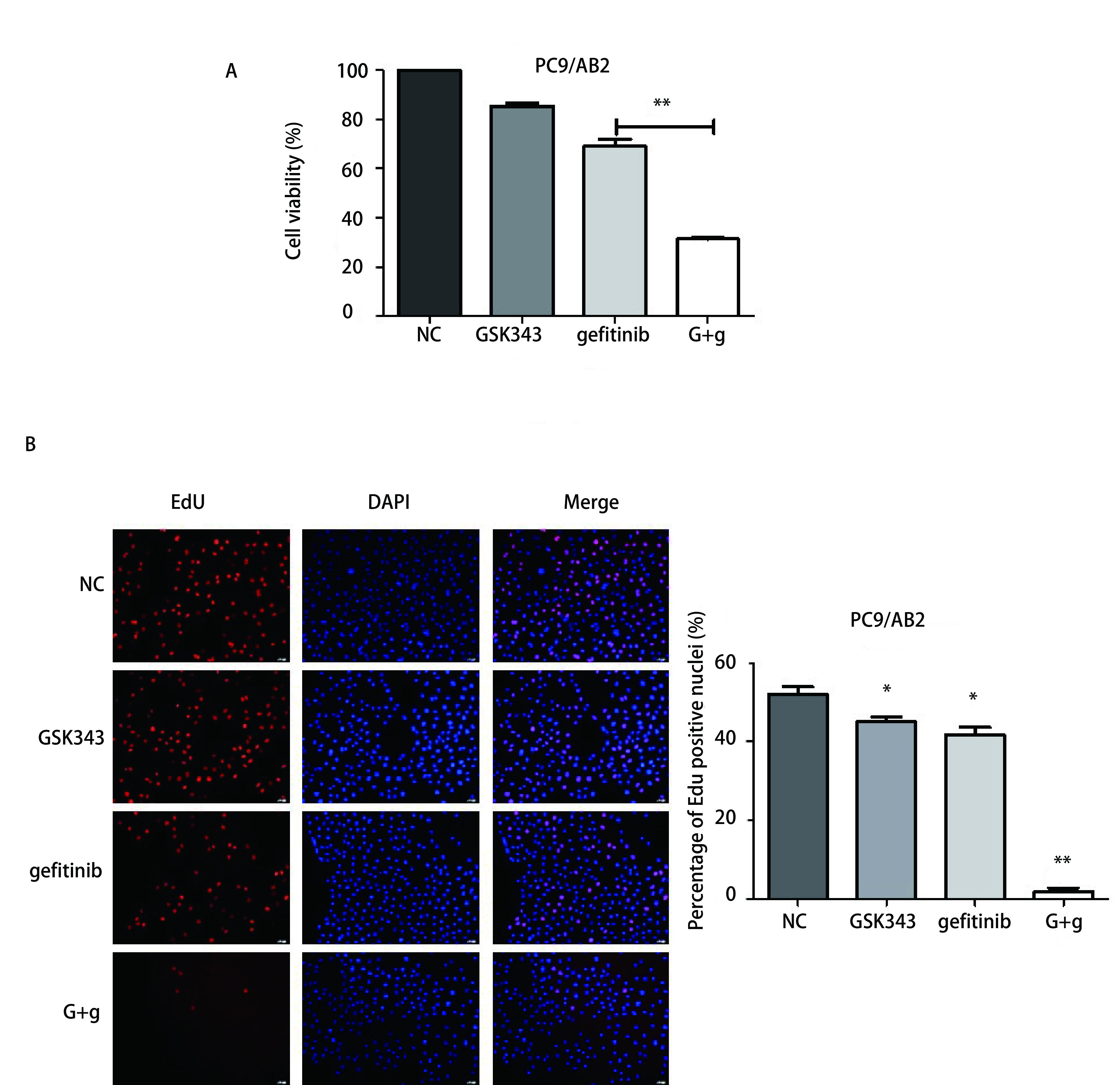
联合应用GSK343+吉非替尼能够显著抑制耐药细胞的活性及增殖。A：CCK-8实验检测细胞活性；B：EdU实验检测细胞增殖 GSK343 combined with gefitinib significantly inhibited cell viability and proliferation in gefitinib-resistant cells. A: CCK-8 assays analysis the cell viability treated with GSK343+gefitinib; B: EdU assays analysis the cell proliferation treated with GSK343+gefitinib.

### 2.3 联合应用GSK343+吉非替尼能显著抑制PC9/AB2细胞的迁移

为了进一步研究联合用药对耐药细胞的迁移作用的影响，我们将实验分为4组：NC阴性对照组（含10%血清培养基）、GSK343组（8 μmol/L）、gefitinib组（15 μmol/L）及联合用药组（8 μmol/L GSK343+15 μmol/L gefitinib），通过细胞划痕实验检测联合用药对于耐药细胞PC9/AB2的迁移能力的改变，分别以16 h及32 h为时间节点。如图 3A所示：用药16 h后，联合应用GSK343+gefitinib（划痕宽度占比为74.59±1.58）较NC阴性对照组（29.18±2.88）能够显著地抑制划痕愈合（*P* < 0.01），单用GSK343（51.36±8.58）也能够对于细胞迁移有一定的抑制作用（*P* < 0.01），而单用gefitinib（35.47±0.83）对于细胞的迁移能力影响较弱，但也有统计学意义（*P*=0.02）；在用药32 h后，NC对照组（1.22±1.17）划痕已经基本愈合，单用GSK343（19.6±1.51）或gefitinib（5.32±1.04）较NC对照组对细胞迁移有一定的抑制作用（*P* < 0.01, *P*=0.01），联合应用GSK343+gefitinib作用效果更为显著（51.52±5.07, *P* < 0.01）。

**3:**
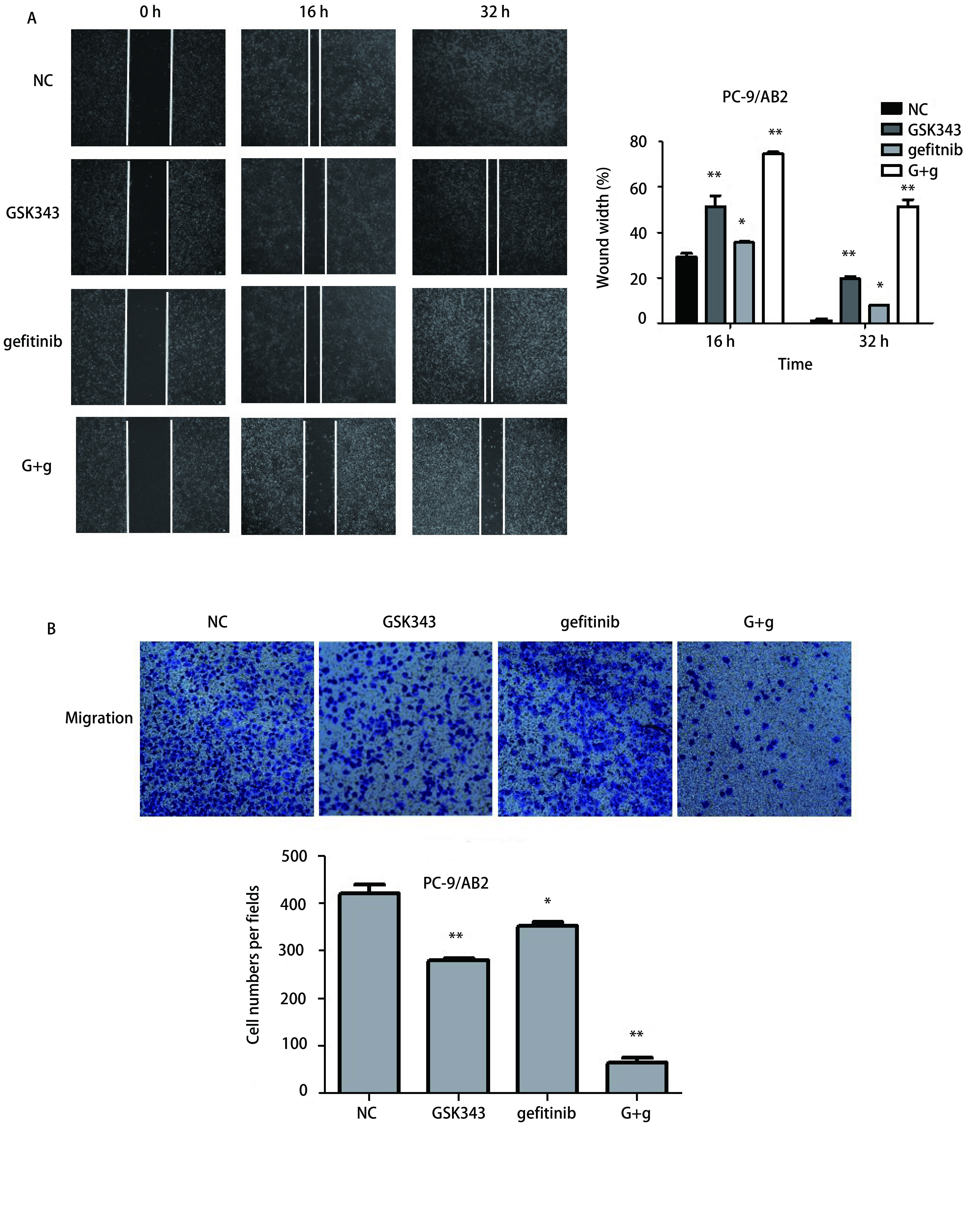
联合应用GSK343+吉非替尼能够显著抑制耐药细胞的迁移能力。A：细胞划痕实验检测细胞的迁移能力；B：Transwell小室检测细胞的迁移能力。 GSK343 combining with gefitinib significantly inhibited the migration ability of gefitinib-resistant lung cancer cells. A: Wound healing assays analysis the cell migration treated with GSK343+gefitinib; B: Transwell chamber assays analysis the cell migration treated with GSK343+gefitinib.

随后，我们分别将细胞分为4组进行不同处理：NC组、GSK343组（9 μmol/L）、gefitinib组（15 μmol/L）、G+g组（9 μmol/L GSK343+15 μmol/L gefitinib），处理时间为48 h，将处理后的细胞转入Transwell小室中，观察24 h细胞迁移至下室的细胞个数，结果显示：GSK343组、gefitinib和G+g组迁出的细胞个数分别为（279.33±8.14）、（352.33±13.65）、（66±14.93），较对照组（420±36.06）；GSK343、gefitinib对细胞的迁移均有一定抑制效果（*P*值分别为 < 0.01、0.038）；G+g联合应用能够显著抑制细胞迁移（*P* < 0.01）。提示联合应用GSK343+gefitinib能够显著地抑制耐gefitinib的PC9/AB2细胞的迁移能力（图 3B）。

### 2.4 联合应用GSK343+吉非替尼能显著诱导PC9/AB2细胞的凋亡

如图 4所示，单独使用GSK343=9 μmol/L处理48 h后细胞，凋亡细胞占总细胞的比例为（5.17±0.93）；单独使用gefitinib=15 μmol/L，处理细胞48 h，凋亡细胞占总细胞的比例为（8.54±0.76）；联合使用GSK343（9 μmol/L）+gefitinib（15 μmol/L），处理细胞48 h，凋亡细胞占总细胞的比例为（32.42±1.04）μmol/L，较NC阴性对照组（1.92±0.34）。GSK343及gefitinib对于耐药细胞能够产生一定的凋亡作用（*P*=0.01, *P*=0.00），但两药联合诱导效果更为显著。

**4:**
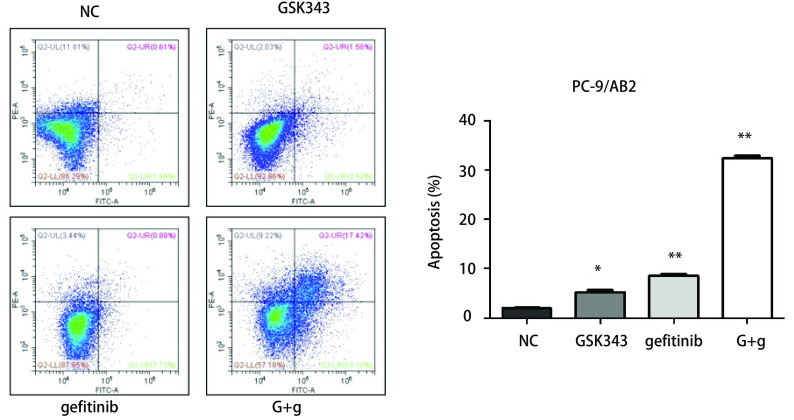
联合应用GSK343+吉非替尼能够显著诱导耐药细胞产生凋亡。 GSK343 combining with gefitinib significantly induced apoptosis of gefitinib-resistant lung cancer cells.

### 2.5 联合应用EZH2抑制剂GSK343+吉非替尼对EGFR信号通路的影响

EGFR-TKIs类药物主要通过抑制EGFR磷酸化从而发挥作用，而EZH2抑制剂主要通过抑制组蛋白H3第27位赖氨酸三甲基化[Tri-Methyl-Histone H3 (Lys27), H3K27me3]从而抑制下游靶基因的表达。我们发现联合应用EZH2抑制剂GSK343+吉非替尼后，ezh2、P-EGFR及H3K27me3的蛋白表达水平较单药组明显降低，提示EZH2抑制剂GSK343联合EGFR-TKI能够显著抑制PC9/AB2细胞EZH2、H3K27me3及EGFR磷酸化（图 5）。

**5:**
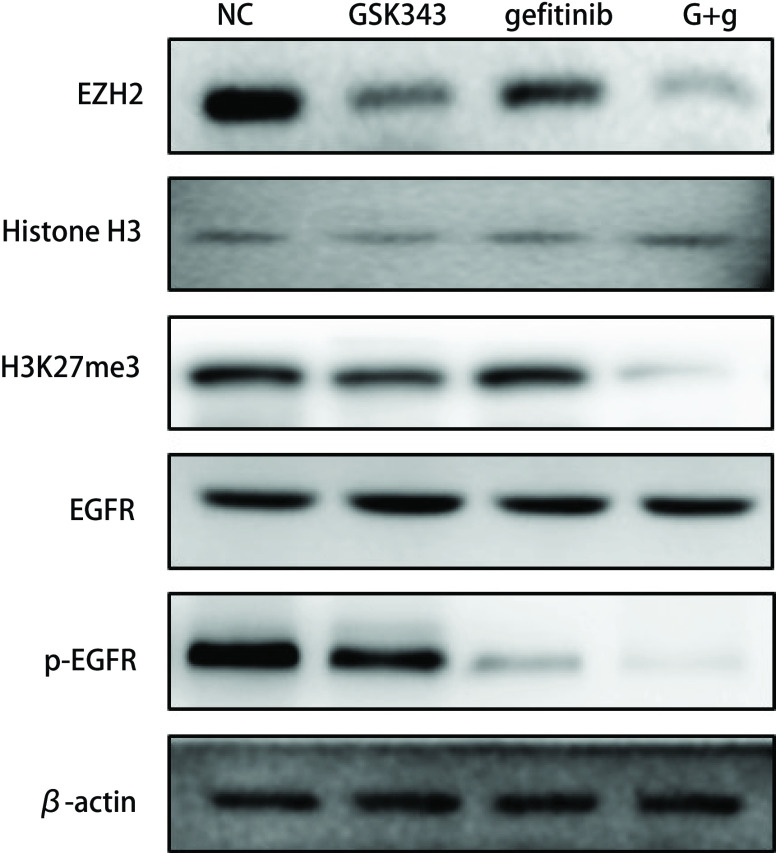
联合应用GSK343+吉非替尼能够显著抑制EZH2、H3K27me3及P-EGFR的表达。 GSK343 combining with gefitinib significantly inhibited the expression of EZH2, H3K27me3 and P-EGFR.

## 3 讨论

分子靶向治疗给肺癌治疗的带来了一场革命性的变化^[12]^。与传统的化疗相比，EGFR-TKIs治疗*EGFR*突变的晚期NSCLC具有效率更高，不良反应更小，治疗后的PFS更长，已成为一线治疗的首选^[13, 14]^。*EGFR*基因突变具有多种类型，且突变类型与EGFR-TKIs的治疗效果相关。85%-90%患者EGFR敏感性突变发生在19外显子缺失或21外显子点突变，这类患者再应用EGFR-TKIs治疗能够显著延长客观缓解率（objective response rate, ORR）及PFS^[15]^。但应用EGFR-TKIs治疗会面临一个严峻的问题即绝大多数肺癌患者会在治疗后1年左右出现耐药^[16]^。因此，开发新型EGFR抑制剂、逆转EGFR-TKIs获得性耐药、及延长药物的耐药周期成为目前EGFR-TKIs研究领域的主要热点。

随着表观遗传学的发展，研究人员发现一些表观遗传学调控相关的复合物在NSCLC中也存在改变或者转录异常，比如EZH2^[17]^。EZH2已经被证实在NSCLC中高表达且与预后相关^[18, 19]^。EZH2抑制剂能够提升肿瘤细胞对抗肿瘤药物的敏感性，增强药物的疗效：应用EZH2抑制剂能够有效地提升具有*BRG1*和*EGFR*突变的NSCLC细胞对依托泊苷的敏感性^[20]^；也有研究表明应用EZH2抑制剂能够通过*PUMA*基因从而逆转NSCLC对于铂类化疗药物的耐药性^[21]^。同时EZH2抑制剂也能够显著抑制细胞的侵袭和迁移能力，EZH2抑制剂能够通过抑制NSCLC中的金属蛋白酶组织抑制因子-3（tissue inhibitor of metalloproteinase-3, TIMP-3）从而抑制癌细胞迁移^[11, 22]^；也有研究证实EZH2抑制剂也能够通过抑制核外调节蛋白talin的直接甲基化从而影响细胞粘附和迁移^[23]^。综上所述，EZH2有可能成为NSCLC的治疗新的靶点，同时EZH2抑制剂也有望给肺癌的治疗带来新的方向。

本实验中联合应用EZH2抑制剂GSK343与吉非替尼，结果显示两药联合应用能够显著地抑制耐药细胞的活性、增殖及迁移，并诱导细胞凋亡，并且能够增强EGFR-TKIs对于EGFR的磷酸化抑制效果。
